# Assessing the impact of grief on quality of life, work productivity, and health outcomes for parents bereaved from SMA: A study protocol

**DOI:** 10.1186/s12962-023-00465-5

**Published:** 2023-08-23

**Authors:** Abigail G. Riley, Christina M. Mulé, Debra Lerner, Lisa Belter, Colleen McCarthy O’Toole, Stacey Kowal, David Fox, Sheila Shapouri, Tamara Vesel, Tara A. Lavelle

**Affiliations:** 1 Center for the Evaluation of Value and Risk in Health, Institute for Clinical Research and Health Policy Studies, Tufts Medical Center, Boston, MA USA; 2 Department of Pediatrics, University of Rochester Medical Center, Rochester, NY USA; 3 Department of Pediatrics, Tufts Medical Center, Tufts University School of Medicine, Boston, MA, USA; 4 Program on Health, Work and Productivity, Institute for Clinical Research and Health Policy Studies, Tufts Medical Center and Tufts University School of Medicine Department of Psychiatry, Boston, MA, USA; 5 Cure SMA, Elk Grove Village, IL USA; 6 Genentech, Inc, South San Francisco, CA USA; 7 Division of Palliative Care, Department of Medicine, Tufts Medical Center, Boston, MA USA

**Keywords:** Grief, Health-related quality of life, Work productivity, Spinal muscular atrophy, Mental health

## Abstract

**Background:**

U.S. cost-effectiveness recommendations suggest that analyses should include all costs and effects relevant to the decision problem [1]. However, in many diseases, including spinal muscular atrophy (SMA), few studies have evaluated bereaved family outcomes after a child has died, neglecting potential impacts on their health-related quality of life (HRQoL), work productivity, and mental health. Additionally, grief-related outcomes are rarely included in economic evaluations. This manuscript outlines the protocol of a study that will estimate the HRQoL, work functioning, and mental health of bereaved parents of children with SMA type 1 to determine how outcomes vary based on parent’s sex and the time since a child’s death.

**Methods:**

This study will involve two phases. In Phase 1, we will conduct a literature review to identify prior research that has measured how parental grief impacts HRQoL, work productivity, and mental health. We will also interview four bereaved parents of children with SMA type 1, stratified by parent sex and time since their child’s death, and analyze findings using a thematic analysis. In Phase 2, we will develop a survey draft based on Phase 1 findings. Parents bereaved from SMA type 1 will review our survey draft and we will revise the survey based on their feedback. We will send a cross-sectional survey to approximately 880 parents bereaved from SMA type 1. We will analyze findings from the survey to investigate whether the severity of grief symptoms is correlated with HRQoL, productivity, depression and anxiety symptom severity. We will also evaluate whether the mean scores of grief and each of the outcomes vary significantly when stratified by parent sex and the time since the child’s death.

**Discussion:**

Our results will provide preliminary information on how parental grief can impact HRQoL, productivity, and mental health outcomes over time. Increasing the availability of family outcomes data will potentially assist organizations performing health economic evaluations, such as the Institute of Clinical and Economic Review (ICER) in the U.S. This research will also help to inform the development of future economic guidelines on this topic.

## Background

With the rising costs of health care, health economic evaluation is increasingly being used by decision makers to guide the use of cost-effective care. Cost-effectiveness analysis (CEA) is one type of economic evaluation that compares an intervention’s incremental costs to its incremental benefits. U.S. guidelines for health economic evaluations specify that when these analyses are conducted from a societal perspective, all costs and health outcomes associated with an illness or intervention should be captured, including those to both the patient and family. Family members, including those who are directly involved in a patient’s care, as well as those who are not involved in care but who share an emotional bond with the patient, can benefit from a patient’s treatment in one or more ways, including through improvements in their health-related quality of life (HRQoL), work productivity, or decreases in caregiving time and out-of-pocket expenses. Current economic evaluation guidelines from Canada [2], the United Kingdom [3, 4], and the Netherlands [5] also recommend the inclusion of these family spillover effects, when relevant. For example, the National Institute for Health and Care Excellence (NICE) recommends incorporating caregiver time in economic evaluations if caregiver services could have been provided by the National Health Service instead. They also recommend incorporating caregiver outcomes if a particular disease or illness has shown to have a substantial impact on a caregiver’s HRQoL [3]. In addition, U.S. guidelines recommend that evaluations be of “…sufficient length to capture all costs and effects relevant to the decision problem”[1]. This may include family direct and indirect costs and health outcomes before and after the death of an individual.

While there is a growing body of research quantifying family outcomes, and an increased use of these outcomes in CEAs, most of these studies have focused on the impact of a patient’s illness on a family member when the patient is alive [6, 7]. However, grief from the death of a loved one can have a significant impact on a family member’s health and functioning. Thimm et al. (2020) found that severe grief in adulthood, which they defined as intense emotional pain, yearning for a loved one who died, and difficulty accepting a loved one’s death, increased the prevalence of mental illness, including depression and anxiety, and these outcomes could persist for many years [8]. While grief can impact all family members, most studies of family grief and associated outcomes following the death of a child have focused on parents. In one study that asked parents to indicate whether they had worked through their grief four to nine years after their child’s death, parents who indicated that they had not resolved their grief reported more sick leave, sleep problems, and hospital visits, than parents who reported that their grief had been resolved [9].

Despite evidence that grief can have a large impact on parent wellbeing, there is scant research that demonstrates changes in outcomes typically used in CEAs, including parent HRQoL, work productivity, and health care costs, and even fewer have included these estimates in evaluations [10, 11]. Among relevant research, two studies found that parental bereavement was associated with lower health utility values, which are quality of life weights used in economic analyses for the purposes of estimating quality-adjusted life years (QALYs). Utilities are reported on a zero to one scale, where zero is equivalent to dead and 1.00 is equivalent to full health. Jong Im Song et al. (2012) found that bereaved parents in Korea (n = 353) had significantly lower utility values (poorer HRQoL) than a matched group of non-bereaved parents, two to six months following the death of a child to cancer (n = 353); (0.88 vs. 0.93, p < 0.01) [12]. Jieun Song et al. (2010) found that, 20 years after the death of a child, utility values for bereaved parents (n = 233) remained lower than those of non-bereaved parents (n = 229); (0.78 vs. 0.82, p < 0.01) [13].

Other studies that have focused on work productivity have found that bereaved parents suffer from short but not long-term productivity loss. In a sample of bereaved parents surveyed 6 months after the death of their child (n = 252), Fox et al. (2014) estimated that parents’ mean absenteeism costs totaled $8,774 (95% CI: $7,795–$10,444) while mean presenteeism costs were $9,638 (95% CI: $8,335–$10,941), in 2011 U.S. dollars [14]. However, in a study comparing parents five years after the death of a child from cancer (n = 42) to non-bereaved (n = 152) parents, Wikman et al. (2016) found no significant differences in employment status or work absences [15].

Limited data on how grief impacts parent HRQoL and costs could be one reason why researchers rarely incorporate these outcomes into economic evaluations. To our knowledge, only one CEA, submitted by a manufacturer and reviewed by NICE to assess Strimvelis for severe combined immunodeficiency caused by adenosine deaminase deficiency, incorporated grief-related HRQoL outcomes into the study’s sensitivity analysis [16]. While accounting for grief made the cost-effectiveness ratio more favorable, it did not change the conclusion. It is unclear whether this is because grief did not have a large enough impact on health and economic outcomes to make a difference, or because the model did not include all relevant grief outcomes (e.g., it included HRQoL but did not include out-of-pocket costs and work productivity loss). Additional grief-related outcomes should be measured so that health economic experts can use these outcomes to assess whether grief should be incorporated into CEAs.

To provide additional data to encourage the exploration of grief-related outcomes in CEAs, this study will measure outcomes among bereaved parents of children with spinal muscular atrophy (SMA) type 1. SMA is a rare, genetic, neurodegenerative disease that causes loss of muscle control and ability to eat, breath, sit, and walk [17]. There are five types of SMA, with type 1 being the most common [18]. There is currently no cure for SMA and prior to the availability of SMA treatments, the average life expectancy of an infant with type 1 SMA was under two years [19]. Over the past seven years, the U.S. Food and Drug Administration (FDA) has approved several new therapies for individuals with SMA type 1, including Evrysdi (risdiplam), Zolgensma (onasemnogene abeparvovec-xioi), and Spinraza (nusinersen). These therapies can improve or preserve motor function and extend survival. However, these therapies are expensive, with Zolgensma having the highest list price of any drug in the U.S. as of May, 2022 [20]. Despite their clinical benefit, ICER value assessments have shown that Zolgensma and Spinraza may not be cost-effective at their current prices across commonly accepted thresholds [21]. However, grief-related outcomes were not included in these evaluations. It is important that future economic evaluations of new therapies include the full range of costs and benefits to appropriately measure the value of new therapies, which may include grief-related outcomes.

We will conduct a multiphase study to measure how parental grief following a child’s death from SMA type 1 impacts a parent’s HRQoL, work productivity, and health. The goal of this paper is to describe how we will measure the impact that grief has on health and economic outcomes. Additionally, findings from our survey can provide guidance for future research measuring the impact of grief on similar outcomes in other disease areas. We will engage stakeholders, including parents, clinical experts, and SMA patient advocacy organization representatives throughout the process to ensure that the study will provide results that are meaningful to the SMA community as well as those working in the field of health technology assessment.

## Methods/Design

### Overview

This study will involve two phases: (1) background research to inform survey design and (2) survey development, data collection and analysis. In Phase 1, we will conduct a literature review on how family grief impacts HRQoL, work productivity, health, and relationships. We will then conduct four semi-structured interviews with bereaved parents of children with SMA type 1 to gather additional information on their grieving experiences. In Phase 2, we will develop and administer a survey to parents who have a child who has died from SMA type 1. We will develop an initial survey draft based on themes that emerged from the literature review and qualitative interviews. The survey will include validated and original questionnaires to ensure that we capture all relevant outcomes for parents who have a child who has died from SMA. We will then conduct cognitive interviews with parents who have a child who has died from SMA type 1 to ensure that the survey is easily understood and has content validity. We will revise the survey before administering it to parents based on feedback from the cognitive interviews. We will analyze the survey data to evaluate how grief impacts HRQoL, work productivity, and health for parents bereaved from SMA, and how the impact varies across time. This survey will provide preliminary HRQoL and productivity data to incorporate into economic evaluations.

### Study Team

The multidisciplinary study team has expertise in health economics, psychology, employee health and work productivity, survey research, and palliative care. For both study phases, we will also collaborate with a Stakeholder Advisory Committee (SAC) to ensure that the findings from the study reflect their understanding of grief for parents who have a child who has died from SMA type 1, and that the study results are meaningful and accessible to the SMA community. The SAC will consist of two parents who have a child who has died from SMA, one mother and one father, one palliative care clinician, one bereaved father with doctoral-level training in grief, and one representative from Cure SMA, an advocacy organization for patients and families affected by SMA. SAC meetings will occur regularly throughout the study and members will provide feedback on study methods and results. SAC guidance will be particularly important as there has been little prior research conducted on SMA-related grief.

### Sample identification and recruitment

We will collaborate with Cure SMA to recruit participants for each phase of the study. Cure SMA is the largest SMA patient advocacy group with 36 chapters throughout the U.S [22]. Its members include individuals and families impacted by SMA as well as clinicians and researchers. Cure SMA maintains a database of parents of living or deceased children with SMA. Cure SMA will email parents bereaved from SMA type 1 with an invitation to participate in qualitative interviews, cognitive interviews, and the survey.

Parents will be eligible to participate in the study if they are the biological, adoptive, or step-parent of a child who died from SMA type 1, are 18 years of age or older, are able to speak English fluently, and are a U.S. resident. We will only recruit parents who are U.S. residents because non-U.S. residents may have other contextual factors that can impact their grief outcomes and should have a separate survey that is developed with these differences in mind. For the qualitative and cognitive interviews, we will only interview one parent from each family to ensure that we are capturing a variety of different experiences and to ensure that our unit of analysis is consistent. However, we will allow both parents from each family to participate in the survey. We will screen parents before the qualitative interviews, cognitive interviews, and survey to determine if they meet the inclusion criteria. We will not offer an incentive to participate in the qualitative or cognitive interviews, because prior research has found that some adults volunteer for interviews because they wish to share their own personal story [23]. During our cognitive interviews, we will determine whether an incentive would be appropriate for the survey.

### Human Ethics

The Tufts Health Sciences Institutional Review Board will review and approve the study protocol and materials prior to the start of each phase of the study.

## Phase 1: background research

### Literature Review

We will first conduct a literature review aimed at understanding how grief impacts HRQoL, work productivity, health, and relationships of bereaved parents. We will also review the literature on the psychosocial outcomes of siblings of children who have died. Because we expect limited literature for grief outcomes related to a child’s death from SMA, we will expand our search to include child deaths from all chronic illness. We will exclude prenatal and suicide deaths, as grief can be substantially different in these circumstances and might have a different impact on HRQoL and work productivity outcomes. The SAC will review our findings and offer feedback on the similarities and differences in experiences of parents bereaved from other chronic illnesses compared to those bereaved from SMA.

### Qualitative interviews

We will interview four parents bereaved from SMA type 1 to supplement findings from the literature review and identify SMA specific grief outcomes to include in the survey. The four parents in our study sample will vary by parent sex and the time since their child died. We will interview three mothers whose children died at different time frames: less than five years ago, five to ten years ago, and over ten years ago. This will provide insights for how grief outcomes may vary since the time of their child’s death. We will also interview one father whose child died five to ten years ago to see how grief outcomes vary based on the parent’s sex. Based on previous literature, these two variables can influence parental grief outcomes.

We will develop a semi-structured interview guide based on the literature review and SAC feedback. Portions of the interview guide will be informed by the conceptual model developed by Snaman et al. (2020) for measuring grief outcomes for parents bereaved from cancer [24]. This model highlights key risk and protective factors that impact grief outcomes, including parent demographics (i.e., parental age, race, and income), characteristics related to the child’s illness and treatment (i.e., length of illness and trust in the care team), and the circumstances surrounding the child’s end of life (i.e., child’s age at death and quality of life). Our interview process will gather data about many of these risk and protective factors including family structure, parent decisions about their child’s care, and the circumstances leading up to their child’s death. We will then ask parents about their experience after their child’s death, including the impact that grief had on their wellbeing, work functioning, and relationships with others.

We will ask the SAC to review the interview guide to ensure that the questions are relevant to parents bereaved from SMA type 1 and are sensitive to the difficult topics to be explored. After the interview guide is finalized, we will conduct 1-hour interviews over Zoom. The interviews will be led by a research faculty member and a research assistant will also be on the call to take notes. Prior to the interview we will ask parents for consent to record the interviews so that we are able to transcribe and code the interviews after we meet with the parents. Parents will be told at the beginning of the interview that they can withdraw from the study at any time and that they can skip any questions that they prefer not to answer. We will provide a peer support contact for parents in case they experience distress after participating in the interviews and would like a resource for support.

After the four interviews are completed the faculty member and research assistant present during all interviews will perform a thematic analysis and start by documenting a list of unique a priori themes within a provisional codebook that follows a similar format to the semi-structured interview guide. The research assistant will then transcribe and deidentify the interviews. Using the preliminary codebook, the faculty member and research assistant present during the interviews will independently code each interview. They will then meet to compare each coded interview, and revise interview codes until a consensus is reached. The research assistant and faculty member will iteratively revise the codebook to include new codes and modify code definitions as new themes emerge. Following coding, we will summarize themes that appear in the interviews, and present the results to the SAC for feedback.

## Phase 2: Survey Development and Fielding

### Planned Survey Design

Based on the findings from the literature review and qualitative interviews, we will develop a survey to measure outcomes associated with grief. As currently planned, the survey will collect information on participants’ background and demographics, and use several validated questionnaires to measure grief, HRQoL, work functioning and productivity, mental health, and physical health.

### Background and demographics

The survey will ask participants about themselves and their child or children who have died from SMA. We will use these demographic data to describe the sample and investigate whether certain parent and child characteristics impact grief outcomes. Demographic questions will include parent sex, age, race, ethnicity, household income, highest level of achieved education, place of residence, and whether the parent was the primary wage earner when their child died. We will also ask parents about the age of their child at the time of their death, the types of interventions their child received during their life, and whether the child received an SMA specific treatment. Finally, we will ask parents about their family and household structure, including the number and ages of individuals who live in their home.

### Grief

We will use the Prolonged Grief Disorder Questionnaire-Revised (PG-13-R) to capture the current intensity of participants’ grief symptoms. Prolonged grief disorder is characterized by continuous grief symptoms that do not decrease over time and impacts an individual’s ability to function [25]. The PG-13-R is a 13-item scale that asks responders to rate the intensity of 13 grief symptoms on a scale from one to five. Individuals who have a score of 30 points or higher, 12 months after the death of a loved one, may have prolonged grief disorder. We will investigate whether the severity of grief symptoms captured in the PG-13-R is associated with levels of HRQoL, productivity, and mental health symptom severity [26]. The PG-13-R was chosen over other grief measures because it focuses on capturing symptoms specific to grief, including longing, disbelief, and loss of identity, rather than symptoms that overlap with other health outcomes like depression and anxiety, which will be measured using separate instruments. The PG-13-R includes a final question that measures whether a study participant’s symptoms of grief impact their current level of social and occupational functioning, but does not include questions to measure past grief outcomes. We will add a question that follows a similar structure to the question about current functioning to also measure whether the symptoms have impacted these areas of functioning in the past.

### HRQoL

We will use the 12-item Short Form Survey version 2 (SF-12v2) to measure HRQoL [27]. The SF-12v2 is a 12-item scale that is commonly used to measure HRQoL for inclusion in economic evaluations [28, 29]. SF-12v2 scores include component scores for both mental and physical health and can be converted into health utility values. We chose The SF-12v2 over other HRQoL scales like the EQ-5D-3L, EQ-5D-5L, or Health Utilities Index Mark 3 (HUI-3) because the SF-12v2 includes domains that are more sensitive to changes in mental health [30]. The SF-12, and the longer version of this instrument, 36-item Short Form Survey (SF-36), have previously been used to measure HRQoL in grieving family members and have shown decreased mental health among the bereaved [31, 32].

### Work Functioning and Productivity

We will use the Work Limitations Questionnaire (WLQ) and the WLQ Time Loss Module (TLM) to capture work productivity [33]. The TLM measures absenteeism while the WLQ measures presenteeism. Absenteeism refers to time missed from working due to physical or mental health problems or care. The TLM captures absenteeism by asking responders to record the number of times they missed a full or half day of work over the past two weeks. Presenteeism refers to an individual’s reduced work function due to physical or mental health problems or care. The WLQ measures presenteeism by asking responders questions about the degree of difficulty performing specific job tasks over the past two weeks. These tasks are common to many jobs. They are also related to objectively-measured work productivity. While both the TLM and the WLQ are not specific grief measures, they are sensitive to a wide range of symptoms that can arise due to many different causes, including grief. We will use both instruments to capture the full impact that grief can have on work functioning and productivity.

### Mental Health

We will use the General Anxiety Disorder Questionnaire (GAD-7) and the Patient Health Questionnaire (PHQ-9) to measure anxiety and depressions outcomes, respectively [34, 35]. These scales are commonly used in clinical practice and measure the frequency of anxiety and depression symptoms. The PHQ-9 and GAD-7 include established cut points for determining symptom severity. The GAD-7 includes thresholds for minimal, mild, moderate, and severe anxiety while the PHQ-9 severity levels are minimal, mild, moderate, moderately-severe, and severe depression. These thresholds will help us measure how mental health differs across different study sub-populations that vary in terms of the time since their child’s death.

### Physical Health

We will use questions in the 2022 National Health Interview Survey (NHIS) as a guide for measuring physical health conditions [36]. Study participants will be asked whether they currently are living with illnesses such as cardiovascular conditions, diabetes, cancer, muscle or joint pain, and alcohol or drug dependence. Previous research suggests that grief can lead to worse long-term physical health outcomes [9]. We will compare chronic illness prevalence in bereaved populations to estimates in non-bereaved populations from previously published sources.

If validated questionnaires do not fully capture SMA and grief-related outcomes, our research team will draft survey questions to supplement preexisting questionnaires. We will ask the SAC to review the draft survey along with alternative instrument measures to ensure that our selected instruments best capture bereaved parent outcomes.

### Cognitive interviews

After we draft the survey, we will interview six parents to provide feedback on whether the survey is clear and reflects their experience as a parent whose child has died from SMA. Interviews will take place over Zoom and will be one hour long. During the interview, parents will read and answer survey questions out loud, and the research team member will ask parents to identify any questions they find difficult or confusing, including where the instructions are unclear. We will also ask them whether the answers and choice options apply to the parent’s experiences. Due to the sensitive subject area, we will also ask parents whether any questions should be rephrased or whether any survey sections are too burdensome. We will spend most of the interview focusing on questions that our study team drafted, as we will not be able to amend any of the validated surveys. However, we may ask a sample of parents to review validated survey instruments to ensure that they do not have any general concerns with a particular survey instrument. We will revise the survey iteratively throughout the cognitive interviewing process to ensure that all revisions are tested before the survey is distributed to the larger SMA community. During the cognitive interviews, we will ask parents whether they believe an incentive would be appropriate for the survey.

### Data Collection

We will program the survey online in REDCap (Research Electronic Data Capture), a secure, HIPAA-compliant software used to collect patient data electronically [37, 38]. Cure SMA will distribute the survey to approximately 880 parents in their database whose child died from SMA type 1 and meet the additional inclusion criteria. The recruitment email will include a link to the survey. Cure SMA will send two reminder emails over a 3-week fielding time.

### Survey Analysis

We will analyze the survey data to estimate grief, HRQoL, work functioning and productivity, mental health, and physical health outcomes in the study sample, and measure how these outcomes vary based on the time since the child’s death. We will stratify time since the child’s death into three categories: less than five years, five to ten years, and greater than ten years. We will calculate descriptive statistics for all demographic and clinical characteristics for the parent, child, and household. We will report categorical variables as counts and percentages, and continuous variables as means and standard deviations.

We will score all validated questionnaires according to standardized procedures and summarize total and, when relevant, component scores using means and standard deviations. When relevant, we will calculate the count and percentage of the sample that fall within pre-defined categories of the scored questionnaire (e.g., mild, moderate, and severe categories of anxiety).

We will compare the prevalence of mental health and physical illnesses in the sample to age-matched population rates using chi-square tests. We will also compare the mean HRQoL scores, utility values, and productivity estimates to population norms using z-tests [29, 39].

We will investigate whether the severity of grief symptoms captured in the PG-13-R is correlated with levels of productivity, HRQoL, and mental health symptom severity using Pearson or Spearman correlation coefficients, depending on the distribution of the variables. We will also evaluate whether the score distributions of the grief, HRQoL, productivity, anxiety and depression outcomes vary significantly when stratified by the time since the child’s death and parent sex. For outcome variables that are approximately normally distributed, we will compare means using the analysis of variance (ANOVA), and will use the Kruskal-Wallis test for skewed distributions. To control for variables that may confound the relationship between time since death and parent outcomes, we will use adjusted regression analyses. We will run five regression models, with grief, HRQoL, productivity, anxiety, and depression being an outcome in each model. We will choose the appropriate regression model based on the distribution of the outcome variable. We will use the time since the child’s death as the main predictor variable, and control for parent, child, and household characteristics. Control variables may include parent age, sex, marital status, race, ethnicity, education, household income, number of living children, number of children who have died, child’s age at death, and hospice or palliative care involvement in child’s death. We will choose the appropriate number of control variables based on the total sample size of respondents.

## Discussion

There are currently no health technology assessment (HTA) guidelines or recommendations on whether family outcomes associated with grief should be incorporated into CEAs. This could be in part due to limited evidence on how grief impacts family health and economic outcomes, and limited experience incorporating these outcomes into CEAs. The results from this study will produce preliminary data on HRQoL and work productivity outcomes for parents bereaved from SMA. These data can be incorporated into economic evaluations to illustrate how including family grief can impact CEA results.

There are several study limitations. First, we expect some selection bias in the qualitative and survey data that may impact the internal validity of our study as parents’ willingness to participate in the study may be related to their current wellbeing. For example, we may be more likely to recruit parents with higher functioning because parents who have had a very difficult time with grief may not be ready to share information about their experiences and may be less likely to participate in the study. This could mean that we would underestimate the mean effect of grief on bereaved parents. However, it is also possible that parents who had a particularly difficult experience might be more likely to want to share their experience, in which case we may overestimate the impact of grief on bereaved parents. In our recruitment email we will highlight the importance of this research as a way to incentivize parents with a range of experiences to participate. Potentially also adding to our selection bias, we will use a patient advocacy group to recruit parents, which may not have a representative selection of parents of children who have died from SMA. Additionally, we will allow, but not require, both parents from the same family to participate in the survey. This may impact our standard errors as parents from the same family may have similar outcomes, but we will not be able to account for clustering since our survey will be anonymous. Finally, since this study investigates a rare disease, there will be limited research to refer to when evaluating our study results and no comparative data from a nationally representative sample for parents bereaved from SMA. This may impact the external validity of our results. Therefore, our findings should be interpreted with caution and should be seen as preliminary findings for how SMA type 1 related grief may impact different outcomes important for economic evaluations.

In addition, due to timeline and resources constraints we chose to focus on grief-related outcomes for SMA type 1 only. However, grief outcomes may vary for parents depending on what SMA type their child had, and the results from our study will likely not be generalizable for other types of SMA. For example, the early onset of symptoms and potential short life expectancy for children diagnosed with SMA type 1 may cause SMA type 1 parents to grieve the life that their child could have lived without their disease. This type of grief may not be present in other SMA types, where the onset of symptoms is later and life expectancy is longer. Additionally, SMA type 1, particularly untreated illness, can require more intensive treatment due to more severe symptoms. This could impact grief outcomes as parents who feel that their child had poor quality of life at the end of life have been shown to have worse grief outcomes.

One goal of this work is to assess how outcomes of parents may vary based on time since their child’s death. However due to study timeline and resource constraints, we have designed a cross-sectional study and will only capture grief outcomes for each parent at one point in time. As a result, we will only capture a potential correlation between grief and time since death, and we cannot determine whether time has a causal impact on grief outcomes. We will control for possible confounders, which are other variables that could have an influence on grief besides the time since death, but it is likely that there are unobserved confounders that we will not be able to control for. Additionally, we will ask parents to answer several questions related to their child’s care prior to their death and recall bias could impact results for parents whose child died several years prior to the administration of the survey. As a result, this study will only provide preliminary data on grief outcomes and patterns of grief over time. As a next step, we will conduct a longitudinal study to provide a clearer picture on the trajectory of grief over time.

Our survey will also be limited by the instruments available to measure HRQoL. Current preference-based HRQoL scales have a focus on health and functioning, and focus less on domains more closely related to grief including loneliness, purpose, and identity. We plan to use the SF-12v2 instrument, because it includes several domains related to mental health, it can capture health utility scores without being too burdensome to complete, and responses can be converted into SF-6D health utility values. We will examine known group differences to determine whether the mental health component of the SF-12, and overall health utility values, can distinguish between caregivers with and without mental illness, including moderate or severe anxiety, or major depression [31, 32, 40]. However, given the potential limitations of this instrument, we may underestimate the full impact of grief on HRQoL outcomes. Underestimating the full impact of a condition, and thus not fully capturing the benefit of treatment, can result in treatment being undervalued in economic analysis.

A group of researchers from the EuroQol Group are currently developing the EQ Health and Wellbeing instrument (EQ-HWB) that may measure outcomes more suitable for capturing grief including a “relationships” category to measure loneliness, social engagement, stigma, support, belonging and connectedness; a “feelings and emotions” category to measure sadness, worry, and hopelessness; an “autonomy” category, to measure coping and control; and a “self-identity” category to measure self-worth [41]. Additionally, the ICEpop CAPability measure for Adults (ICECAP-A) is an instrument that measures feelings of security; love, friendship and support; independence; achievement and progress; and enjoyment and pleasure; that may also be more sensitive to grief outcomes and can be incorporated into economic evaluations [42]. While the EQ-HWB and ICECAP-A both include domains that may better capture grief when compared to HRQoL scales, we chose to use the SF-12v2 because we had limited survey space, and it can produce health utility values and QALYs. However, future research should measure grief-related outcomes using the EQ-HWB and ICECAP-A to fully capture the range of domains that are relevant to wellbeing and capability in this population.

Our results will provide preliminary information on how parental grief can impact HRQoL and productivity outcomes. This manuscript outlines one potential way to measure family spillover effects related to grief and bereavement to encourage the exploration of grief outcomes in economic evaluations. Collecting this data will be important as many HTA organizations, including the Institute for Clinical and Economic Review (ICER) in the U.S., have stated their intention to include the full range of family spillover effects in their value assessments that are conducted from a societal perspective [43], but can only do so if data are available. This research will also help to inform the development of future guidelines on this topic.

## Data Availability

Not applicable.

## Abbreviations

CEA: Cost-effectiveness analysis
HRQoL: Health-related quality of life
QALY: Quality-adjusted life year
NICE: National Institute for Health and Care Excellence
SMA: Spinal muscular atrophy
SAC: Stakeholder Advisory Committee
PG-13-R: Prolonged Grief Disorder Questionnaire-Revised
SF-12: Short Form Survey
HUI-3: Health Utilities Index Mark 3
WLQ: Work Limitations Questionnaire
TLM: Time Loss Module
GAD-7: General Anxiety Disorder Questionnaire
PHQ-9: Patient Health Questionnaire
NHIS: National Health Interview Survey
REDCap: Research Electronic Data Capture
ANOVA: Analysis of variance
HTA: Health technology assessment
EQ-HWB: EQ Health and Wellbeing Instrument
ICECAP-A: ICEpop CAPability measure for Adults
ICER: Institute for Clinical and Economic Review
